# Prevalence, associated risk factors and antimicrobial susceptibility pattern of *Campylobacter* species among under five diarrheic children at Gondar University Hospital, Northwest Ethiopia

**DOI:** 10.1186/1471-2431-13-82

**Published:** 2013-05-21

**Authors:** Ayalew Lengerh, Feleke Moges, Chandrashekhar Unakal, Belay Anagaw

**Affiliations:** 1 College of Medicine and Health Sciences, University of Gondar Teaching Hospital Laboratory, Bacteriology Section, University of Gondar, Gondar, Ethiopia; 2 Department of Medical Microbiology, School of Biomedical and Laboratory Sciences, College of Medicine and Health Sciences, University of Gondar, Gondar, Ethiopia

**Keywords:** Antimicrobial susceptibility, *Campylobacter* species, Under five children, Risk factors

## Abstract

**Background:**

Recent reports indicate that *Campylobacter* species are becoming one of the leading causes of bacterial diarrhoeal disease worldwide and most of the isolates are resistant to different antibiotics. This study aimed at determining the prevalence, associated risk factors and susceptibility pattern of *Campylobacter* species in under-five diarrheic children.

**Methods:**

A cross-sectional study was conducted from October 2011 to March 2012. Samples were collected from under five diarrhoeic children who visited University of Gondar Teaching Hospital and seeking medical services during the study period. Stool specimens were aseptically inoculated using selective media and species isolation was further processed following standard procedures. Antimicrobial susceptibility test for *Campylobacter* species was performed using the standard agar disc diffusion method. The data was entered and analyzed using SPSS version 16 packages. Odd ratio was used to see their association between variables and then logistic regression was used to measure strengths of association. *P-values* less than 0.05 were taken as statistically significant.

**Result:**

A total of 285 under five children with diarrhoea were included in this study. Of these144 (50.5%) were males and 141(49.5%) were females with the age range of one month to five years and mean age of 2.26 years (25months). Among 285 stool specimens cultured, 44(15.4%) of them were positive for *Campylobacter* species. Culture positivity for *Campylobacter* was higher in children below 12 months of age. Latrine usage, water source, boiling drinking water, bottle feeding, nutritional status and exposure to domestic animals had statistically significant association. Highest drug resistance rate were found in ampicillin (68.2%), tetracycline (56.8%) and trimethoprim- sulfamethoxazole (54.5%).

**Conclusion:**

Isolation rate of *Campylobacter* species were frequent among under five children. The frequency was higher in those children who were malnourished, drinking of unprotected water and direct contact with infected animals (especially cats, dogs, pigeons, hens and their products). The antimicrobial resistance patterns for some of the commonly prescribed antibiotics were high. Therefore, awareness of hand washing and proper boiling of drinking water are probably important in preventing infection with *Campylobacter* species and childhood diarrhea should not be underestimated and effectiveness of the drugs should be continuously monitored by doing antimicrobial susceptibility test.

## Background

*Campylobacter species (Campylobacter spp.)* are small gram-negative, non-spore-forming, helical bacteria with a distinctive ‘darting’ motility, and are catalase and oxidase positive. *Campylobacter spp*. can be found in the reproductive organs, intestinal tracts, and oral cavity of animals and humans [1]. Diarrhoeal diseases are common in children aged less than five years, consumption of contaminated water and food is the major source of infection. Among *Campylobacter* spp., most commonly isolated species from cases of gastroenteritis was *Campylobacter jejuni (C. jejuni*) followed by *Campylobacter coli*. More recently, other *Campylobacter* species have been recognized as gastrointestinal pathogens in both industrialized and developing countries [2]. *Campylobacter* spp. are leading cause of bacterial diarrhoeal disease worldwide resulting mainly from contamination of poultry, or other meats, raw milk and milk products and surface or raw water [3].

The increasing rate of human infections caused by anti-microbial resistance strains of *Campylobacter* makes clinical management of cases of campylobacteriosis more difficult. Anti-microbial resistance can prolong the illness and compromise treatment of patients with bacteraemia. The rate of anti-microbial resistant enteric infections was highest in the developing world, where the use of anti-microbial drugs in humans and animals are largely unrestricted [4].

In Africa, a few studies have indicated that campylobacteriosis is most common among children of young age. In Ile-Ife, Nigeria, *C. jejuni* was found to be an important agent of diarrhoea in children [5]. In Durban, South Africa, *Campylobacter* were found in 21% of diarrhoeal cases among children aged less than five years [6]. Again in Venda, South Africa, *Campylobacter spp*. were also isolated from 20% of stool samples tested from HIV-positive individuals [7].

In Ethiopia, studies have revealed that diarrhoeal diseases are major causes of infant and child mortality and morbidity. About 39,000,000 episodes of diarrhoea per year were estimated to occur in Ethiopia; out of which 230,000 deaths occur in children below five years of age [8]. The pediatric admission review at Jimma hospital showed that diarrhoea was the second leading cause of admission and hospital deaths and *Campylobacter* is one cause of diarrhoea in the area [9]. There is no recent report on *Campylobacter* spp. and their drug susceptibility patterns in the North western Ethiopia. Therefore, this study aimed at determining the prevalence, associated risk factors and antimicrobial susceptibility pattern of *Campylobacter* spp. causing enteritis in under five diarrheic children at Gondar University Hospital, Northwest Ethiopia.

## Methods

### Study design and period

A cross-sectional study was conducted in Microbiology Laboratory at University of Gondar Teaching Hospital between October, 2011 and March, 2012. It is referral hospital that provides services to over 5 million inhabitants in the Northwest, Ethiopia. All the under five diarrhoeic children coming to the pediatric ward of the Gondar hospital seeking for treatment during the study period were the source population.

### Sample size determination and Sampling technique

A total of 285 samples were collected from under five diarrhoeic children who visited University of Gondar Teaching Hospital and seeking medical services during the study period. Convenient sampling technique was used.

#### Inclusion criteria

All the under five diarrhoeic children coming to the pediatric ward of the Gondar hospital seeking for treatment during the study period.

#### Exclusion criteria

Those diarrheoic children under five years who had treatment with antibiotics in the last 5 days were excluded.

### Data collection procedure

After obtaining written consent from the guardian, data about the socio-demographic characteristics, associated risk factors and relevant clinical information were taken using pre-structured questionnaire by pediatrician. To assess the validity of the questionnaire pre-test was conducted at the polyclinic: one of the health centers found in Gondar town. Two laboratory technologists were responsible to process the stool specimen for isolation of *Campylobacter* spp. To ensure the reliability of the information, the guardian’s were interviewed in their local language. All the questionnaires were checked for its completeness and consistency every day.

### Specimen collection and processing

Fresh stool specimen was collected aseptically from each study subject using sterile screw-capped containers and transported immediately to the Microbiology Laboratory, University of Gondar. Specimens were inoculated on *Campylobacter* Agar Base (Karmali) (Oxoid, Ltd,England) supplemented with sodium pyruvate, cefoprazone, vancomycin and cyclohexamide then, kept in a 2.5 liter anaerobic jar and Campy-Gen gas generating kit (5% O_2_ and 10% CO_2_) (Oxoid CN0025A) was inserted to maintain the microarophilic condition. The jars were incubated at a temperature of 42°C for 48 hrs. The identification of *Campylobacter* spp was performed by characteristic appearance on culture medium (moist, creamy-grey and flat-spreading), gram stain, oxidase test, catalase reaction and dry spot *Campylobacter* test (Oxoid, Basingstoke, Hampshire, England). The type strains *C*. *jejuni* (LMG 13646) was inoculated as positive control.

### Antimicrobial susceptibility test

Antimicrobial susceptibility test for *Campylobacter* spp was performed using the standard agar disc diffusion method as recommended by Clinical and Laboratory Standards Institutions (CLSI). The commonly prescribed antimicrobials were obtained from Oxoid at the concentration of ampicillin (30 μg), amoxicillin with clavulanic acid (30 μg) gentamicin (10 μg), tetracycline (30 μg), doxycycline (30 μg), chloramphenicol (30 μg), ciprofloxacin (5 μg), norfloxacin (5 μg), ceftriaxone (5 μg) erythromycin (15 μg) clindamycin (15 μg) and trimethoprim-sulphamethoxazole (25 μg). In brief, 3–4 morphologically identical colonies of bacteria from culture were picked and suspended in sterile normal saline. Turbidity of the broth culture was compared with that of 0.5 McFarland turbidity standards (10). A loop full of the bacterial suspension was placed at the center of Muller Hinton agar media (Oxoid, LTD) supplemented with 5% sheep blood and evenly spread using sterile cotton tipped applicator. After drying, antibiotic discs were placed and incubated at 42°C for 48 hours in anaerobic jar using CO_2_ generating kits. Finally, the diameter of growth inhibition around the discs was measured and interpreted as sensitive (S), and resistant (R) as per the guidelines of the manufacturer. Control strains of *E. coli* (ATCC 25922) sensitive to all antibiotic being tested was inoculated to evaluate the performance of culture media and antibiotic discs.

Susceptibility tests to naldixic acid (30 μg) (Oxoid, UK) and Cephlotin (30 μg) (Oxoid UK) were performed for all isolates of *Campylobacter spp*. in accordance with the criteria set by the National CLSI using the disk diffusion method [10]. The isolates were classified as sensitive and/or resistant according to the standardized tables supplied by the CLSIs. *Campylobacter* strains that were sensitive to naldixic acid but which are resistant to Cephlotin were considered *C. jejuni* and *C. coli*, while strains that were resistant to both drugs were considered other species [11].

### Data processing and analysis

The data was entered and analyzed using SPSS version 16 packages. Odd ratio was used to compare association between *Campylobacter* spp and other variables of the study. Logistic regression was also used to assess associations with dependent and independent possible risk factor. *P-*values less than 0.05 were taken as statistically significant.

### Ethical consideration

The study was conducted after obtaining institutional ethical clearance from Research and Publication office of University of Gondar. Permission was taken from Gondar University Hospital administrators and written consent also obtained from the guardians of study subjects. Positive study subjects to *Campylobacter* spp. were referred to the physician with their result for treatment.

## Results

### Socio-demographic characteristics

A total of 285 under five children with diarrhoea were included in this study. Of these144 (50.5%) were males and 141 (49.5%) were females with the age range of one month to five years and mean age of 2.26 years (25months). Seventy three (25.6%) of them were younger than one year and 212 (74.4%) were in the age range of 1 to 5 years. Majority of them were urban dwellers 230(80.7%) while 55 (19.3%) were rural dwellers [Table 1].

**Table 1:** **Distribution of *Campylobacter *infection in under five children with diarrhoea at Gondar University Hospital, Northwest Ethiopia, October 2011 to March 2012**

**Variables**	**Campylobacter species**				
	**Positive**	**Negative**	**Total**	**OR (95% CI)**	***P*-value**
	**N (%)**	**N (%)**	**N (%)**		
**Age in year**					
<1	15(34.1)	58(24.1)	73(25.6)		
1-5	29(65.9)	183(75.9	212(74.4)	1.63 (0.77, 3.42)	**0.16**
**Sex**					
Male	21(47.7)	123(51)	144(50.5)		
Female	23(52.3)	118(49)	141(49.5)	0.88 (0.44,1.75)	**0.69**
**Residence**					
Urban	34(77.3)	196(81.3)	230(80.7)		
Rural	10(22.7)	45(18.7)	55(19.3)	0.78 (0.34, 1.83)	**0.53**

*OR* = Odds ratio, *N*: total number of under five children.

### Prevalence of *Campylobacter* species

Among 285 stool specimens cultured, *Campylobacter* species were isolated from 44 (15.4%), from which 40/44 (90.9%) were *C. jejuni* and *C. coli* and 4/44 (9.1%) were other species. Twenty one (14.6%) of the male and 23(16.3%) of the female children were positive for *Campylobacter* species. Although, the variation was not statistically significant, the culture positivity for *Campylobacter* was relatively higher for children below 12 months of age compared to other age groups (*P =* 0.16). Even though, most study subjects live in urban, culture positivity rate is relatively higher in rural with the percentage of 14.8% and 18.2%, respectively [Table 1].

### Possible risk factors and their association with *Campylobacter* infections

Among the risk factors caretaker relation to child, education level of caretaker, family size, washing hands before feeding and preparing foods, cleaning utensils with soap and hypochlorite, washing the child with soap and water after defecation showed no statistically significant association with *Campylobacter* culture positivity; whereas usage of latrine, source of water, boiling drinking water, bottle feeding, nutritional status and exposure to domestic animals had statistically significant association [Table 2]. Of the 58 family who do not use latrine always 15(25.9%) of their children were found to be positive for *Campylobacter* species. Family who used latrine always were less likely to be positive for *Campylobacter* infection than those who do not use (AOR = 0.42; CI = 0.2, 0.90; *P =* 0.01).

**Table 2:** ***Campylobacter *infection and associated risk factors in under five diarrheic children at Gondar University Hospital, Northwest Ethiopia, October 2011 to March 2012**

**Risk factors**	**Positives**	**Negatives**	**Total**	**COR**	**95% CI**	**AOR**	**95% CI**	***P*-value**
	**N (%)**	**N (%)**	**N (%)**					
**Caretaker relation to child**								
Mother	38(15.2)	212(84.8)	250(87.7)	1		1		
Father	4(28.6)	10(71.4)	14(4.9)	2.23	0.56,8.38	2.2	0.66,7.48	0.29
Guardian	2(9.5)	19(90.5)	21(7.4)	0.59	0,09,2.78	0.58	0.11,2.62	
**Education of caretaker**								
Illiterate	14(12.1)	102(87.9)	116(40.7)	1		1		
Primary	15(19.2)	63(80.8)	78(27.4)	1.73	0.73,4.12	1.45	0.78,3.83	0.20
Junior-secondary	13(20.6)	50(79.4)	63(22.1)	1.89	0.77,4.61	2.7	0.82,4.33	
Diploma & above	2(7.1)	26(92.9)	28(9.8)	0.56	0.08,2.85	3.9	0.7,16.0	
**Number of children**								
Only 1	7(15.6)	38(84.4)	45(15.8)	1		1		
2-5	32(14.7)	185(85.3)	217(76.1)	0.94	0.36,2.53	0.92	0.38,2.28	
>5	5(21.7)	18(78.3)	23(8.1)	1.5	0.42,5.40	1.3	0.42,5.40	0.73
**Latrine usage**								
No	15(25.9)	43(74.1)	58(20.4)	1		1		
Yes, always	29(12.8)	198(87.2)	227(79.6)	0.42	0.21,0.90	0.41	0.20,0.84	0.01*
**Hand washing before feeding**								
Not at all	5(27.8)	13(72.2)	18(6.3)	1		1		
Yes, sometimes	14(15.9)	74(84.1)	88(30.9)	0.49	0.13,1.88	1.9	0.5,6.3	0.52
Yes, always	25(14)	154(86)	179(62.8)	0.59	0.12,1.50	0.06	0.02,0.19	
**Clean the child after defecation**								
Not at all	6(25)	18(75)	24(8.4)	1		1	0.15,1.26	0.29
Yes, sometimes	18(12.9)	122(87.1)	140(49.1)	0.44	0.14,1.44	0.48	0.2,1.68	
Yes, always	20(16.5)	101(84.5)	121(42.5)	0.59	0.19,1.92	0.69		
**Cleaning materials for utensils**								
Water only	10(11.8)	75(88.2)	85(29.8)	1		1		
Soap	33(17.6)	154(82.4)	187(65.6)	1.61	0.71,3.69	1.60	0.70,3.43	0.33
Chemicals	1(7.7)	12(92.3)	13(4.6)	0.63	0.03,5.61	0.62	0.07,5.33	
**Water source**								
Pipe	35(14.1)	214(85.9)	249(87.4)	1		1		0.001*
Well	3(75)	1(25)	4(1.4)	18	1.63,44.4	2	2.0, 20	
Spring	3(12)	22(88)	25(8.7)	0.83	0.2,3.2	0.78	0.2, 2.0	
River	3(42.9)	4(57.1)	7(2.5)	4.5	0.77,25.6	4.8	1.2, 21	
**Boiling water**								
Yes	2(2.4)	81(97.6)	83(20.1)	1	2.4,65.2	1	2.5, 45	<0.001 ^α^
No	42(20.8)	160(79.2)	202(70.9)	10.6		10.57		
**Exclusive breastfeeding**								
No	15(18.5)	66(81.5)	81(28.4)	1	0.35,1.53	1	0.6, 2.7	0.36
Yes	29(14.2)	175(85.8)	204(71.6)	0.73		1.37		
**Bottle feeding**								
No	20(10.8)	165(89.2)	185(65)	1		1	1.36, 5.0	0.008*
Yes	24(24)	76(76)	100(35)	2.61	1.29,5.3	2.60		
**Nutritional status**								
Well nourished	30(12.4)	211(87.6)	241(84.6)	1	1.47,7.31	1	1.58, 6.8	0.002*
Malnourished	14(31.8)	30(68.2)	44(15.4)	3.3		3.2		
**Exposed to domestic animals**								
No	10(7.6)	122(92.4)	132(46.3)	1		1		
Cats and dogs	21(29.6)	50(70.4)	71(24.9)	5.12	2.11,12.7	6.3	2.25,11.65	<0.001 ^α^
Hen and pigeons	8(19)	34(81)	42(14.7)	2.87	0.94,8.69	2.9	1.05,7.88	
Other animals	5(12.5)	35(87.5)	40(14.1)	1.74	0.48,6.05	1.4	0.5,5.4	

*AOR*: Adjusted odd ratio, *COR*: Crude odd ratio, * p-value < 0.05, ^α^ p-value < 0.001.

The culture positive rate of *Campylobacter* species among study subjects who use pipe, well, river, and spring as a source of drinking water were 35 (14.1%), 3(75.0%), 3 (12.0%) and 3 (42.9%), respectively (Table 2). Children who used well and river as a source of drinking water had 4.6 and 2 times (AOR =2.0; CI = 2.0, 20.0; p = 0.001and AOR = 4.59; CI = 1.2, 21; *P* = 0.001) likely to be positive for *Campylobacter* infection, respectively. Children who drank boiled water were more protected from *Campylobacter* infection compared to non-boiled water users (AOR = 10.6; CI = 2.5, 45; *P* = 0.001). Of the one hundred children who use bottle feeding, 24 (24.0%) were positive for *Campylobacter* species compared to non bottle feeders 20/185 (10.8%). This indicate that children who used bottle feeding were 2.6 times (AOR = 2.6; CI = 1.36, 5.0; *P* = 0.008) more affected by *Campylobacter* infections. Malnourished children 14 (31.8%), were three times infected than well nourished 30 (12.4%) (AOR = 3.2; CI = 1.58, 6.8; *P* = 0.002). High culture positive rate of *Campylobacter* species had been observed in children who were exposed to domestic animals compared to non exposed. Children who were exposed to pet animals, hens and pigeons were found 2.9 times (AOR = 2.87; CI = 1.05, 7.88; *P* = 0.001) affected than non-exposed individuals. While children, who were exposed to cats and dogs were 5.1 times (AOR = 5.12; CI = 2.25, 11.65; *P* = 0.001) affected than non exposed children [Table 2].

The main significant clinical presentations for the *Campylobacter* culture positive children were abdominal pain (*P* = 0.002), but other symptoms like, fever, vomiting, duration of diarrhea, stool frequencies per 24 hours and stool consistency were not statistical significant in culture positive and negative patients [Table 3].

**Table 3:** **Association between *Campylobacter *infected children and clinical findings at Gondar University Hospital, Northwest Ethiopia, October 2011 to March 2012**

**Clinical finding**	**Positive**	**Negative**	**Total**	**OR (95% CI)**	***P*-value**
	**N (%)**	**N (%)**	**N (%)**		
**Fever**					
yes	18(40.9)	79(32.8)	97(34)	1.42 (0.72,2.88)	0.29
No	26(59.1)	162(67.2)	188(66)		
**Vomiting**					
yes	29(65.9)	137(56.8)	166(58.2)	1.47 (0.71,3.04)	0.26
No	15(34.1)	104(43.2)	119(41.8)		
**Abdominal pain**					
Yes	41(92.2)	172(71.4)	213(74.7)		
No	3(6.8)	69(28.6)	72(25.3)	5.34 (1.56,22.9)	0.002*
**Duration of diarrhoea**					
1-5 days	40(90.9)	222(92.1)	262(91.9)		
≥6 days	4(9.1)	19(7.9)	23(8.1)	0.86 (0.26,3.15)	0.78
**Stool frequency per day**					
1-4 /day	36(81.8)	196(81.3)	232(81.4)		
≥5/ day	8(18.2)	45(18.7)	53(18.6)	1.03 (0.42,2.59)	0.93
**Consistency**					
Watery	27(61.4)	160(66.4)	187(65.6)		
Mixed (blood and mucus)	17(38.6)	81(33.6)	98(34.4)	0.80 (0.40,1.64)	0.52

*OR* = Odd ratio, * p-value < 0.05.

### Antimicrobial susceptibility patters of the isolates

The results of antimicrobial susceptibility testing for *Campylobacter species* isolated from under five children with diarrhoea against 14 chosen antimicrobial agents are presented in Table 4. Lower resistance rate was observed in naldixic acid (9.1%), followed by chloramphenicol (11.4%) and norfloxacin (11.6%). However, higher drug resistances were observed in ampicillin (68.2%), tetracycline (56.8%) and trimethoprim sulfamethoxazole (54.5%).

**Table 4:** **Drug susceptibility patterns of the *Campylobacter *isolates at Gondar University Hospital, Northwest Ethiopia, October 2011 to March 2012**

**Drugs**	**Susceptibility pattern**			
	**Sensitive**		**Resistant**	
	**N**	**%**	**N**	**%**
Erythromycin	34	77.3	10	27.7
Clindamycin	26	59.1	18	40.9
Trimethoprim sulfamethoxazole	20	45.5	24	54.5
Ciprofloxacin	37	84	7	16.0
Ceftriaxone	34	86.4	10	27.7
Chloramphenicol	39	88.6	5	11.4
Naldixic acid	40	90.9	4	9.1
Cephlotin	5	11.4.	39	88.6
Gentamicin	36	81.8	8	18.2
Amoxicillin with clavulanic acid	28	63.6	16	36.4
Ampicillin	14	31.8	30	68.2
Tetracycline	19	43.2	25	56.8
Doxycycline	37	84.1	7	15.9
Norfloxacin	38	86.4	6	11.6

*N*: number of *Campylobacter* species.

Among the 44 *Campylobacter* culture positives, multidrug resistance (an isolate being resistant to two or more drugs) were detected in 30 (68.2%) of the strains. Among these 16 (36.4%) were resistant to four drugs (Table 5).

**Table 5:** **Multiple drug resistance patterns of *Campylobacter *isolates in under five diarrhoeic children at Gondar University Hospital, Northwest Ethiopia, October 2011 to March 2012**

**Drugs**	**No of *Campylobacter spp *isolated (N = 44)**	**Percent**
R0	11	25.0
R1	3	6.8
R2	6	13.6
R3	8	18.2
R ≥ 4	16	36.4
Total	44	100

R0 = No drug resistance, R1 = Resistant to one drug, R2 = Resistant to two drugs, R3 = Resistant to three drugs, R4 = Resistant to four drugs.

## Discussion

This study showed that the prevalence of *Campylobacter* species in under five children with diarrhoea was 15.4%, which is slightly higher than other findings in Gondar [12], Dembia [13], Bahir Dar [14] and Jimma [15] with isolation rates of 13.8%, 10.5%, 8% and 11.6%, respectively. Lower incidence of Campylobacteriosis has been reported in Cameroon (7.7%) [16], Zimbabwe (9.3%) [17] and Egypt (9%) [18]. On the other hand slightly higher prevalence had been reported from Algeria (17.7%) [19], Nigeria (16.5%) [20], Tanzania (18%) [21], and South Africa (21%) [22] and higher prevalence reported from Bangladesh (26%) and Thailand (41%) [23,24]. This could be due to differences in geographical location, study population, study period and method employed for each study. Our finding is consistent with reports from Iran (14.7%) [25] Peru (15%) [26] and Addis Ababa (15.3%) [13].

The distribution of *Campylobacter* species between females and males was not statistically significant, which agrees with the study results reported in different parts of Ethiopia [12,13,15,27]. Although the finding was not statistically significant, higher rates were observed in rural (18.2%) than urban (14.8%), resident children, which is in line with the findings in Yemen and Mexico [28,29]. This may be due to unprotected water source and presence of domestic animals in almost all rural house hold.

In this study, high infection rates were seen in under five children whose family didn’t use the latrine regularly and those whose family had no latrine in their home. Drinking water from unprotected source like river and well had statistical significant association with *Campylobacter* species culture positivity rate; so boiling drinking water had protective effect against *Campylobacter* infection, which is consistent with the study conducted in Yemen and Colorado [28,30]. Our study also revealed that bottle feeding had also showed significant risk factors to *Campylobacter* infection in under five children. This can be explained by low level sterilization of the bottles, use of unpasteurized milk and storage of cooked food for later use.

High infection rates were seen in children who have close contact with pet animals (cats, dogs), pigeons and hens, which indicate the direct association between *Campylobacter* species infection and pets, as it is already pointed out that direct contact with these animals is a frequent mode of transmission to humans [30]. The rate of culture positivity was more likely higher among malnourished children than well nourished. This is consistent with the previous report in Chile [2], Gondar [12], Jimma [15] and Addis Ababa [27]. This may be due to low immune status of malnourished children.

In this study, abdominal pain was the most common symptom (92.2%) and had statistically significant association with isolation of *Campylobacter* species among under five children. This is consistent with the previous studies conducted in Thailand [18], Yemen [28], Jimma [15] and Addis Ababa [27]. Other symptoms such as fever, vomiting, duration of diarrhea, stool frequency per day and stool consistency were not important symptoms in the present study, which is similar with the study at Dembia [13] but different from previous studies in Thailand and Gondar, Jimma in which watery diarrhoea and the duration of diarrhoea had significant association, respectively [12,15,18].

Even though most gastroenteritis caused by *Campylobacter* species is often a mild and self-limiting disease, it causes mild to severe dehydration and occasionally spreads to the bloodstream and causes life-threatening infections in children; in this case it requires antibiotic treatment. In this study, fourteen antibiotics were tested against 44 isolates of *Campylobacter* species. Most tested isolates were sensitive to naldixic acid (90.9%) chloramphenicol (88.6%), nurfloxacin (86.4%), doxycicline (84.1%) ciprofloxacin (84%) and gentamicin (81.8%). Among these, norfloxacine ciprofloxacin and doxycicline are not prescribed to children due to their contradictions. A study from Jimma reported that all isolates of *Campylobacter* were sensitive to chloramphenicol and gentamicin [15]. This is different from our result that 11.4% and 18.2% resistance were detected in chloramphenicol and Gentamicin, respectively.

*Campylobacter* species were equally resistant to erythromycin and ceftriaxone in this study (22.7%), this is alarming because erythromycin is the drug of choice for *Campylobacter* species, and ceftriaxone it the 3^rd^ generation cephalosporin, which is recently available in the market. Erythromycin resistance was higher than the previous study in Jimma (10%) [15] and Addis Ababa (2%) [13]. This might be due to the fact that erythromycin may act as a selective pressure and favor the proliferation of resistant strains.

A high percentage of amoxicillin with clavulanic acid resistance (36.4%) was also observed. This high rate may be due to indiscriminate use of amoxicillin in the area that leads to increased resistance. The majority of the isolates were resistant to ampicillin and trimethoprim-sulfamethoxazole (68.2%, 54.5%) respectively. This is almost similar to other studies conducted in Jimma and Addis Ababa [15,27]. As it was indicated in another study, this higher resistance could be either because they are commonly prescribed and sold on the open market without prescription [15]. The rate of resistance to tetracycline against *Campylobacter* species (56.8%) in our study was higher than other results like Jimma (14%) [15], Addis Ababa (10%) [27] and Bahir Dar (22.2%) [14]. This might be due to the reason that tetracycline is frequently prescribed and sold in open market without prescriptions.

Compared with other similar research findings conducted in Addis Ababa and Jimma, this study showed increased resistance to most antibiotics. The frequency of multidrug resistant strains (resistant to two or more drugs) (68.2%) in this study was higher than the previous finding in Ethiopia (20%) [15].

## Conclusions

The present study indicates that infection caused by *Campylobacter* species was very frequent among the under five children. The frequency were higher in malnourished than well nourished children. The possible risks of infection to children were through drinking of unprotected water or by direct contact with infected animals (especially cats, dogs, pigeons, hens and their products). Therefore, awareness of hand washing and proper boiling of drinking water are probably important in preventing infection with *Campylobacter* species. Our result showed that the resistance rate of *Campylobacter* species increased through time. This high rate of resistance reflects either frequently prescribing drugs without drug susceptibility testing results or inappropriate usage of the commonly available drugs in the local market. Therefore, providing treatments for children should be based on updated information on susceptibility pattern of *Campylobacter* species rather than clinical symptoms. Therefore, the clinicians and laboratory personnel should consider that diagnosing infection with *Campylobacter* species is as important as other infection with enteric pathogens. Further studies to investigate the role of domestic animals in the transmition of the disease, species differentiations and serotyping of *Campylobacter* spp are recommended.

## Competing interests

All authors declare that they have no competing interest.

## Authors’ contributions

AL conceived the study, participated in sample collection, performed laboratory diagnosis, conducted data analysis and drafted the initial and final draft manuscript, FM, Prepared the initial and final drafts of the manuscript*,* CU, reviewed the initial and final drafts of the manuscript, BA, reviewed the initial and final drafts of the manuscript. All authors read and approved the final manuscript.

## Pre-publication history

The pre-publication history for this paper can be accessed here:

http://www.biomedcentral.com/1471-2431/13/82/prepub
